# Promoting ball-based play and sports participation for preschool children

**DOI:** 10.3389/fspor.2026.1815686

**Published:** 2026-06-10

**Authors:** Lars Breum Christiansen, Trine Top Klein-Wengel, Marlene Rosager Lund Pedersen, Malte Nejst Larsen

**Affiliations:** Department of Sports Science and Clinical Biomechanics, University of Southern Denmark, Odense, Denmark

**Keywords:** ball-based play, early childhood education and care, preschool intervention, sports club collaboration, sports participation

## Abstract

**Introduction:**

This study investigates the effects of a preschool intervention, *Ball-based Play in Preschool*, on two primary outcomes: children's participation in ball-based leisure sports and a composite index of enjoyment, skill level, and frequency of ball-based play. Secondary descriptive analyses examine the distribution of these outcomes and their associations with background characteristics.

**Methods:**

The intervention was developed to integrate ball-based play into daily preschool routines and contained collaboration between preschools and local sports clubs to foster ball-based sports participation. The non-randomized wait-list control design included children from eight preschools whose parents completed a questionnaire assessing child-level outcomes and background factors.

**Results:**

One of the assessed outcomes was the newly developed Ball Play Index capturing three key elements of ball play: frequency, enjoyment and skill level. Baseline data revealed substantial variation across the eight participating preschool settings. For instance, participation in ball-based sports ranged from 14% to 30%, while participation in any sport varied from 31% to 80%. Cross-sectional analyses of 429 responses identified associations between sports participation, the Ball Play Index, and factors such as gender, disability status, and parental background. Impact analyses (*n* = 108) showed no significant effects of the intervention on either Ball Play Index (*β* = −0.07, 95% CI −0.29 to 0.14; *p* = 0.508) or participation in ball-based leisure sports (OR = 1.24, 95% CI 0.22–6.97; *p* = 0.810).

**Conclusion:**

Future initiatives should account for both the readiness of sports clubs and the existing activity levels within the target group. A broader approach that includes diverse sports may help overcome capacity challenges, and ensuring equitable access remains essential. Finally, future efforts may benefit from involving parents more actively in the process.

## Highlights

• Introduction of a new Ball Play Index to assess children's participation, enjoyment and skill level in ball-based play.
• Large baseline variation across preschools suggests contextual factors strongly influence sports participation.
• The preschool intervention actively collaborating with local sports clubs was well received but did not affect formal ball-based sports participation or Ball Play Index.

## Introduction

It is recognized that physical activity during early childhood is important, particularly in promoting healthy developmental outcomes (1, 2). From a holistic perspective, engaging in physical activities is essential not only for physical health but also for fostering social skills, motor development, and emotional well-being (3–5). The preschool years serve as a critical period for establishing foundational movement skills and a positive attitude towards physical activity that can influence lifelong patterns of engagement (6, 7). It is during this early stage that children explore their abilities and develop preferences for specific types of play, which enhance their motor skills and facilitate peer interactions (8, 9).

Recognizing the significance of early exposure to physical activities from a holistic perspective, interventions aimed at increasing participation in ball-based play and activities can be a promising strategy for promoting active lifestyles among young children. Ball-based activities are recognized for their potential to develop a broad range of movement competencies (10, 11). Skills such as throwing, catching, and dribbling strengthen children's object control, a key aspect of movement competence (12). Ball-based skills can be understood as a set of foundational movement skills, reflecting a set of goal-directed movement patterns that support physical activity across the lifespan (6). Beyond physical benefits, participation in ball-based play and activities can potentially foster holistic development due to the integration of cognitive, socio-emotional and motor skill challenging aspects (9, 13). Importantly, it can be tailored to different age groups, such as preschoolers, and promote collaboration and communication (10).

Moreover, the increasing emphasis on preschool children's physical activity and motor skill development has led researchers to advocate for structured play and physical activities, including ball games, as vital components of physical education at this age (7, 14, 15). One priority setting is childcare, where several studies have been conducted to promote physical activity (14, 16). Strategies range from structured lessons to provision of equipment and role modelling (17). To our knowledge, very few studies have included the combination of childcare and leisure time organized sports. One example from Denmark examines how two national ball-sport federations collaborated with preschools to engage parents and increase sports participation (18). The study highlights the importance of preschool–club collaboration in communicating opportunities for organized sport and supporting early engagement among young children.

Preschool-aged children (3–6 years) and their participation in organized sports is an emerging field in the body of international research (19). However, several countries do not include children below 5 years in their national monitoring of sports participation and the research in the field is limited (20). Organized sport during early childhood is related to motor development^1^, and could potentially contribute to children's psychological, social and emotional development (20). Early participation in organized sport may therefore lay the foundation for life long physical activity, even though, the merits of an early start has been questioned (19). A longitudinal register-based study from Australia tracked children participating in modified sport programs and found that many withdrew rather than transitioning into club sport, highlighting challenges in sustaining early participation within organized sport (22). On the other hand, a Finnish longitudinal cohort study of 501 children aged 3–8 years showed that participation in organized sport during early childhood was associated with higher levels of moderate-to-vigorous physical activity and lower sedentary time 3 years later, suggesting potential longitudinal associations between early sport participation and physical activity behavior (23).

Participation rates in organized sports among preschool-aged children differ across countries and are influenced by methodological variations, which should be considered when comparing findings across studies. As an example, a Danish study based on a national survey reported that approximately 80% of the children between 3 and 6 years participated in organized sport, where the three most popular types were gymnastics, swimming and soccer (24). In Australia, 40% of the 0–4-year-olds and 75% of the 5–8-year olds participate in organized sport according to a national population tracking survey (25), witnessing a large increase in participation in the transition throughout early childhood. In a Canadian context, reportedly 46% of children between 3 and 4 years enrolled in organized sport (20), and in Finland non-representative studies report sports club participation rate for 3–8 year old children of about 60% of which one third was ball sports (21, 23). Overall, these data indicate substantial cross-country variation in early sport participation but consistently suggest that a large proportion of preschool-aged children are already engaged in organized sport activities, highlighting the importance of early childhood as a key period for intervention and sport engagement.

In many countries, national sport federations, community-sponsored initiatives, and for-profit organizations have developed modified sports programs by adapting activities and games to better suit younger children (19, 20, 22, 26). In Denmark, sports clubs are primarily non-profit, volunteer-based organizations that play an important role in civil society (27), with high participation rates among children (28). Gymnastics, dance and swimming have traditionally been the most prevalent organized sports for young children in Denmark, but more recently ball-based sports has been adapted to this age group with more focus on play and less on competition as well as involving parent participation (18). This is the case for soccer, volleyball, basketball, badminton, and handball, which are sports included in the intervention investigated in the present study. This earlier start in organized sport can however be uneven as studies show higher participation in middle class families or families with a background in sport (26).

This study investigates the effects of a preschool intervention (*Ball-based Play in Preschool*) on two primary outcomes: children's participation in organized ball-based leisure sports and a composite index capturing enjoyment, skill level, and frequency of ball-based play. Secondary analyses examine the distribution of these outcomes and overall sports participation, and their cross-sectional associations with background characteristics.

## Materials and methods

### Study design and participants

**Figure 1:**
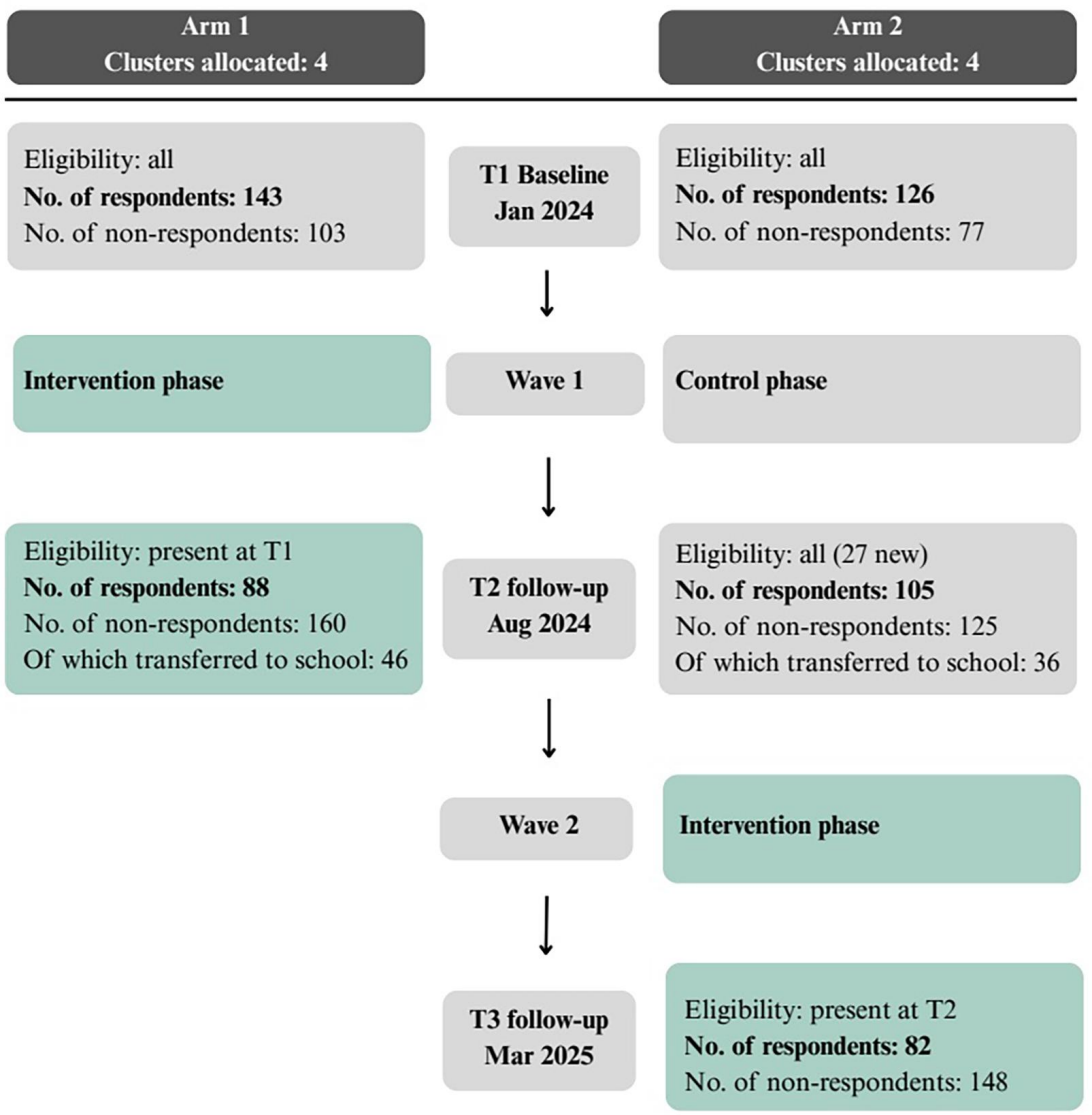
Flow chart of participant eligibility, study arms, and data collection rounds. Sample 1 include parents from preschools in both arms who completed questionnaires at baseline and first follow-up (impact analyses). Sample 2 include parental questionnaires collected across three data collection rounds (cross-sectional analyses).

The study is a non-randomized wait-list control intervention entitled *Ball-based Play in Preschool*. Recruitment was conducted in collaboration with Danish municipalities and the National Olympic Committee and Sports Confederation of Denmark (DIF) through the *Move for Life* partnership (*Bevæg dig for livet*). Four municipalities agreed to participate, each contributing with two preschools and collaborating sports clubs to the subsequent intervention. All children enrolled in the eight preschools at baseline were eligible if parental consent was obtained. Recruitment took place between November 20, 2023, and January 10, 2024. The impact analyses are based on parental questionnaires collected at baseline and first follow up (Sample 1), where the first four preschools had finished the 7-month intervention period (Figure 1). The cross-sectional analyses are based on parental questionnaire collected at baseline, first and second follow up (Sample 2).

### The intervention

**Figure 2:**
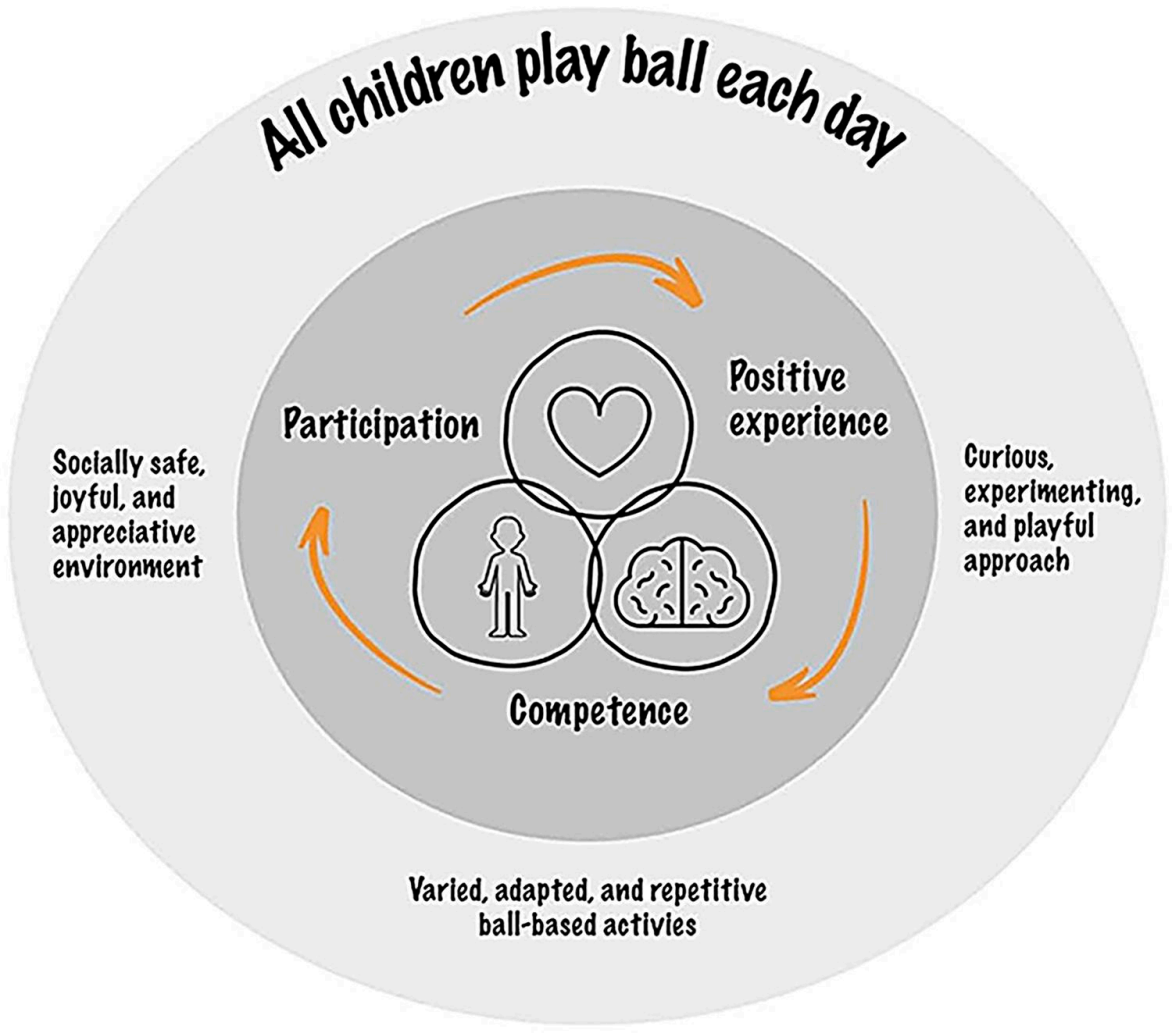
The intervention-model used in the project with the overall aim that all children play ball each day. The intervention-model is developed for the project and inspired by theory related to physical activity promotion (9, 29).

The intervention was developed in collaboration between municipal consultants, lecturers and students from Early Childhood Teacher Education and Physiotherapy Education (UC SYD, UCN, UCL and VIA UC), researchers from the University of Southern Denmark and five national sports federations (handball, soccer, basketball, volleyball and badminton). The project was guided by theory related to physical activity promotion (9, 29) and visualized in a guiding model developed for the study (Figure 2). Drawing on principles of physical literacy (29, 30), the guiding model emphasizes daily engagement in ball-based play and activities for all preschool children. Besides the overall aim of daily ball play, key elements include attention to the child's physical and cognitive abilities, as well as their intrinsic motivation. Furthermore, the model illustrates a positive iterative process where participation, experience, and competence reinforce each other. And finally, secure supportive conditions—such as a safe social environment, a playful approach, and sufficient variation and repetition—help children practice, gain experience, and develop competence. Accordingly, the intervention did not prescribe fixed durations or frequencies of ball-based activities but promoted a flexible, context-sensitive integration into everyday practices and daily routines.

The intervention was initiated through partnerships between preschools, sports clubs, and municipalities, with planning meetings to ensure shared understanding and support. Each preschool appointed 2–5 *champions* who received training and led the integration of ball-based play and activities into daily routines. The champions developed action plans for ball-based activities and acted as links between preschools and sports clubs.^2^

The champions and the majority of sport club coaches attended a 6-hour training course introducing the project principles and selected activities. During the process, project staff visited preschools to observe, support reflection, and plan next steps, combined with ongoing dialogue and follow-up.^3^ Preschools and sport clubs received balls and activity cards (Examples in Supplementary Material), and parents received a booklet and a small ball for each child to promote attention and engagement at home.

A collaboration program between preschools and local sports clubs was established focusing on ball-based play and activities. This initiative aimed to facilitate transition of children into sports clubs, especially focusing on families with low sporting or economic capital. The program had a planned duration of eight visits at either sport facilities or at the preschool involving all children, coaches and preschool staff. Additionally, a concluding event named, *ball festival*, was organized as part of the program at each site. The evaluation of the collaboration program is reported in Danish elsewhere (31).

### Parental questionnaire

A parental questionnaire was developed to assess background characteristics and child-level outcomes. The questionnaire was pilot tested with experts and parents and revised following cognitive interviews (32). Data was collected via online questionnaire (SurveyXact) with researchers assisting parents at preschools to increase response rates. Besides the questions related to the child-level outcome the questionnaire included items related to the following background characteristics: age, gender, disability, language spoken at home, hours in preschool, economy and parents' experience with leisure sport (Table 1).

**Table 1:** Sample characteristics.

Variables	Sample 1		Sample 2
	Intervention (*n* = 56)	Control (*n* = 52)	All (*n* = 465–544^a^)
Age (*M*, SD)	4.1 (0.8)	3.9 (0.6)	4.6 (0.9)
Gender (girls %)	51.8	46.2	48.5
Disability (%)	5.7	10.0	5.7
Hours per week in preschool (*M*, SD)	35.1 (6.5)	35.3 (5.1)	35.0 (6.2)
Native speaking at home (%)	67.3	78.9	68.3
Very sport experienced parent (%)	52.0	38.2	42.6
Perceived good economy (%)	61.1	54.0	52.7
Leisure time sport (%)	66.1	46.2	55.5
Leisure time ball-based sport (%)	32.1	13.5	21.9
Ball Play Index—BPI (M, SD)	4.0 (0.6)	3.6 (0.7)	3.9 (0.7)
Perceived frequency (daily and weekly %)	69.6	51.0	65.0
Perceived experience (very fun and fun %)	96.4	76.0	89.0
Perceived skill (very confident and confident %)	70.9	55.1	71.2

a *n* in Sample 2 refers to observations; analyses include repeated measurements from 372 parents.

#### Participation in leisure-time ball-based sports

Participation in sport during leisure was the first child-level outcome assessed with the yes/no question: *These questions relate to whether your child plays sports in a club in their leisure time. Does your child attend (1) Ball games (e.g., sandbox ball, ball and movement, badminton, soccer, handball), (2) Gymnastics, motor skills, rhythm, dance or similar, (3) Swimming, (4) Other sports*. For the impact analyses only participation in ball-based sports were included, while participation in other sports is reported and cross-sectionally analyzed.

#### Participation, enjoyment and skill level related to ball-based play

The second child-level outcome was based on theoretical perspectives of children's participation and development in physical activity (2, 29) and directly linked to the intervention model (Figure 2). Parents were asked: *(1) How often does your child play with balls at home? (from every day to never), (2) How does your child experience playing with balls? (from very funny to very boring), and (3) How confident does your child seem in its abilities when playing with balls (e.g., throwing, catching and kicking)? (from very confident to very unconfident).*

As described above, the questionnaire was pilot tested with experts and parents and refined through cognitive interviews to ensure clarity, relevance, and interpretability. The three items capture complementary dimensions of children's engagement in ball play (frequency, enjoyment, and perceived competence), supporting the construct validity of the combined index. The items were measured on ordinal Likert-type scales with different response formats and were therefore standardized prior to aggregation to ensure comparability across items. The Ball Play Index was constructed as a composite score using equal weighting of the standardized items, based on the theoretical assumption that frequency, enjoyment, and perceived competence represent equally important and interrelated dimensions of children's engagement in ball-based play. Internal consistency of the three items was acceptable (Cronbach's *α* = 0.70). An exploratory factor analysis showed that a single factor accounted for most of the variation, with factor loads ranging from 0.59 to 0.69. Based on this, the *Ball Play Index* (BPI) was calculated as the mean of the three items, providing a reliable summary measure of children's everyday participation, enjoyment, and skill level in ball play. Item-level descriptive statistics, including means, standard deviations, and response distributions, are presented in Table 1 and Supplementary Figures S1–S3.

#### Background characteristics

Some categorical responses were recoded into dichotomous variables: parent sports experience (very experienced vs. some, less and none experience), economic situation (good vs. moderate and poor) and home language (only Danish vs. other and mixed). Hours in preschool were calculated multiplying the usual number of days per week with the usual number of hours. Questions on disability and gender had dichotomous response options, while age was treated as a continuous variable.

### Analyses

The impact analyses were conducted as pre–post analyses on complete cases with data available at both baseline (T1) and at 7-months follow-up (T2) (Sample 1: intervention and control). To ensure comparability, the impact analyses were restricted to the first intervention wave, where a concurrent control group was available. In addition, cross-sectional association analyses were performed using all available data from T1, T2, and T3 (Sample 2: full sample).

Linear mixed models were used for continuous outcomes, and mixed-effects logistic regression models for binary outcomes. All impact models included fixed effects for group, time, and their interaction (group × time), as well as age and sex, with random effects for child and preschool. The group × time interaction estimated the intervention effect. This approach accounts for baseline differences by modelling within-individual changes over time. Association analyses used mixed-effects logistic regression models including age, time, sex, and relevant covariates (disability, language background, socioeconomic status, and self-efficacy), with random effects for child and preschool. No interaction terms were included. All analyses were performed using Stata BE version 18.

### Ethics and data management

Parents and children received information about the study's purpose, voluntary participation, and data handling procedures. Written informed consent was obtained from all participating parents. Data were treated confidentially, accessible only to the research team, and stored in accordance with regulations from the Danish Data Protection Agency. All participants remain anonymous in publications. In line with Danish law (LBK no. 1338 of 1/9/2020) and review by the Ethics Committee of Southern Denmark (case: 20232000-77), formal ethical approval was not required as no biological material was collected. The study was approved by the Research and Innovation Organization (RIO) at the University of Southern Denmark (review no. 11.944).

## Results

First, we present the participants in the two analyzed samples and distribution of organized sports participation. This is followed by the cross-sectional analyses, with the impact analyses concluding the section.

Table 1 presents characteristics of Sample 1, consisting of the intervention group (*n* = 56) and the control group (*n* = 52), and Sample 2 (*n* = 465–544, depending on the variable) used in the cross-sectional analyses. The 108 participants in Sample 1 answered before and after the first intervention wave. Participants in Sample 1 are included in Sample 2 (2 × 108) together with answers from parents in each of the three rounds, which were not included in Sample 1. Sample 2 comprises repeated measurements across three rounds comprising 372 parents and 544 observations: 233 parents provided data once, 106 twice, and 33 in all three rounds (233 + 2 × 106 + 3 × 33 = 544). A total of 449 children in the eight preschools were invited to participate in the baseline measurements (Figure 1). Of those 367 children were still attending preschool at first follow-up in August and constitute the eligible population for Sample 1. Additionally, 27 children were added to the population of the second wave (preschools in the control group). The total number of eligible children for Sample 2 was 995 children (449 at baseline +367 at first follow-up + 27 added to the second wave +152 follow-up for second wave). Response rate for Sample 1 was 29% and for 47%–55% for Sample 2.

Intervention groups in Sample 1 were generally comparable at baseline regarding age, gender and time in preschool per week. The intervention group had fewer native speaking parents and fewer reported disabilities. They were also more likely to have a very sport experienced parent and to participate in organized sports (66.1% vs. 46.2%) and specifically ball-based sports (32.1% vs. 13.5%). The Ball Play Index (BPI) was higher in the intervention group, which was related to differences in all three questions in the index. Almost two thirds of the children were reported to play ball at least weekly. More parents reported having children playing ball at home more often in the intervention group. The groups also differed in how children experienced ball-based play and their perceived skill level. At baseline, 96.4% of parents in the intervention group and 76.0% in the control group reported that their child found ball-based play very fun or fun. Additionally, 71.2% of parents in the intervention group and 55.1% in the control group believed their child was confident or very confident. Exact distributions of answers for the three questions can be found in Supplementary Figures S1–S3.

The focus of the current study is on ball-based sport and play, but for contextual purposes we report on the distribution of the other sports in the survey. Across all eight preschools and 544 responses (including repeated measurement) 56% of the parents stated participation in organized leisure sports ranging from 31% to 80% between preschools (Table 2). The most reported sport was gymnastics, followed by ball-based sports and swimming. Large variations were observed—especially in gymnastics ranging from 12% to 65%. This wide range highlights the substantial variability between preschools, which is important for understanding the context in which the intervention was implemented.

**Table 2:** Rate of participation in organized leisure sport (*n* = 544).

Sport	Total average	Min.-max preschool average
Gymnastics, motor skills, rhythm, dance or similar,	32%	12%–65%
Ball games (e.g., sandbox ball, ball and movement, badminton, soccer, handball)	22%	14%–30%
Swimming	19%	9%–27%
Other sports	8%	3%–25%
At least one of the above	56%	31%–80%

*n* refers to observations; analyses include repeated measurements from 372 parents.

The cross-sectional regression analyses revealed several associations between background characteristics, the BPI as well as sports participation (Table 3). Age was negatively associated with the BPI (*β* = –0.09, *p* = .022), but the odds ratios for sports participation (OR = 1.56 and 1.57) indicated a non-significant trend toward increased involvement (*p* > .07). Follow-up rounds showed significantly higher BPI compared to baseline across all preschools (*β* = 0.15 and 0.22, *p* < .01), while ORs for sports participation were elevated but not statistically significant, particularly at second follow-up (OR = 1.93, *p* = .302).

**Table 3:** Associations of Ball-Based Play Index (BPI) and participation in organized sport with individual and family factors.

Variable	Ball Play Index (BPI) (*n* = 429)			Participation in ball-based sport (*n* = 429)			Participation in any sport (*n* = 429)		
	*β*	SE	*p*	OR	SE.	*p*	OR	SE	*p*
First follow-up (ref. baseline)	.15	.05	0.002	.94	.39	0.884	1.58	.58	0.212
Second follow-up (ref. baseline)	.22	.08	0.007	1.93	1.23	0.302	1.48	.86	0.498
Age (years)	−.09	.04	0.022	1.56	.45	0.129	1.57	.40	0.077
Girls (ref. boys)	−.18	.06	0.008	.22	.13	0.008	1.85	.83	0.168
Disability (ref. no disability)	−.34	.13	0.008	.13	.17	0.104	.14	.13	0.034
Native speaking at home (ref. foreign speaking)	−.34	.08	0.000	.48	.28	0.211	1.02	.54	0.970
Good economy (ref. medium and poor)	.06	.06	0.351	1.91	.92	0.180	1.62	.68	0.245
Very experienced in sport (ref. medium, low and none)	.26	.06	0.000	2.29	1.16	0.104	2.82	1.27	0.022
Constant	4.49	.21	0.000	.02	.03	0.011	.07	.10	0.050

*n* refers to observations; analyses include repeated measurements from 372 parents. OR, odds ratio; *β*, regression coefficient; *p*, *p*-value; Random effects account for clustering at preschool and child levels (8 preschools, 287 children with 1–3 responses).

Gender differences were evident: girls had significantly lower BPI (*β* = –0.18, *p* = .008) and markedly lower odds of participating in ball-based sports (OR = 0.22, *p* = .008), though their odds of participating in any organized sport were not significantly different from boys (OR = 1.85, *p* = .168). Children with disabilities showed consistently lower engagement across all outcomes, with significant negative associations of BPI (*β* = –0.34, *p* = .008) and organized sports participation (OR = 0.14, *p* = .034). Speaking the native language at home was negatively associated with BPI (*β* = –0.34, *p* < .001) but had no significant effect on sports participation (OR ≈ 1.0, *p* = .970).

Economic situation and prior sport experience showed positive trends. Although perceived good economy was not significantly associated with any outcome (*p* > .18), the ORs for sports participation were relatively high (OR = 1.91 and 1.62). Having a very experienced parent in sport was strongly associated with the BPI (*β* = 0.26, *p* < .001) and with higher odds of participating in any sport (OR = 2.82, *p* = .022). Overall, while not all effects reached statistical significance, several predictors showed consistent directional trends across outcomes.

Table 4 presents the regression analyses of the intervention effects, which focus solely on the hypothesized effect outcomes: ball-based sports participation and BPI. At baseline, children in intervention preschools had significantly higher odds of participating in ball-based sport during leisure (OR = 6.18, *p* = 0.022). No significant main effect of time or intervention × time interaction was observed for this outcome. For BPI, children in intervention preschools had higher scores (*β* = 0.39, *p* = 0.014), and scores increased slightly over time (*β* = 0.23, *p* = 0.009). No significant intervention × time interaction was observed. In the empty model, clustering was relatively low at the preschool level but substantial at the child level for both outcomes, with ICCs of 0.075 and 0.094 for preschool and 0.616 and 0.614 for child-level variation for ball-based sports participation and BPI, respectively. The distributions of answers for the three questions in the BPI are presented in Supplementary Figures S1–S3 across intervention groups and from baseline to follow-up.

**Table 4:** Effects of the intervention on participation in leisure-time ball-based sport and Ball Play Index (BPI).

Outcome	Predictor	Effect	95% CI	*p*	ICC (preschool/child)
Participation in leisure-time ball-based sport	Intervention (ref. control)	OR = 6.18	1.30–29.43	0.022	0.075/0.616
	Follow-up (ref. baseline)	OR = 1.00	0.23–4.32	0.998	
	Intervention × Time	OR = 1.24	0.22–6.97	0.810	
Ball Play Index (BPI)	Intervention (ref. control)	*β* = 0.39	0.08–0.70	0.014	0.094/0.614
	Follow-up (ref. baseline)	*β* = 0.23	0.06–0.41	0.009	
	Intervention × Time	*β* = −0.07	−0.29 to 0.14	0.508	

OR, odds ratio; *β*, regression coefficient; CI, confidence interval; *p* = *p*-value; ICC values calculated from random effects variance components. Random effects account for clustering at preschool and child levels (8 preschools, 108 children with 2 responses).

## Discussion

The overall aim of *Ball-based Play in Preschool* was to develop and evaluate a multicomponent intervention promoting ball-based play and sports participation for all children aged 3–6 years. Interestingly, we found large variation between the eight preschool settings at baseline for outcomes as well as background characteristics. As an example, participation in ball-based sport ranged from 14% to 30% and participation in gymnastics ranged from 12% to 65%. Cross-sectional analyses showed several associations between sports participation, the Ball Play Index (BPI) and background characteristics, i.e., gender, disability, and parental background. These associations are, however, not enough to explain the large variations in sports participation. The impact analyses of the intervention showed no effects for either BPI or participation in ball-based sport. The following section will discuss these findings and explore their implications for the design and implementation of future interventions.

The intervention did not produce significant effects on either children's participation in ball-based sport during leisure or the BPI. Children in intervention preschools started with higher BPI scores and showed a modest increase over time on all three items in the index (Supplementary Figures S1–S3), but the interaction term was non-significant. This baseline difference suggests a potential imbalance between groups, and although the analytical approach accounted for baseline differences, the relatively high baseline levels in the intervention group may have limited the scope for further improvement and made them less responsive to the intervention. The index were developed with the intention of capturing three key elements of physical active play: participation, enjoyment and skill level (2, 29). While enjoyment of ball-based play overall was high, approximately one third of the children played ball less than weekly at home. In the study all children received a small ball and an activity booklet with ideas for home use. Daily participation increased from 21% to 30% in the intervention group. However, greater focus on parental involvement and a stronger emphasis on home-based ball play might have been needed to reach children who remained to play very rarely at home. This highlights the potential of targeted interventions in the home setting—an area where existing evidence remains limited. Few studies have focused specifically on home-based approaches, but those that did showed promising results when parent or provider practices were actively changed, such as integrating movement into daily routines (14, 33).

Participation in ball-based sports was also very different between intervention groups and only minor changes was reported during the 7 month follow-up. This suggests that the multicomponent intervention including the collaboration program was insufficient to shift overall sports participation patterns in the short term. An alternative explanation is that the hypothesized positive iterative process, where participation, experience, and competence reinforce each other, primarily unfolded within the preschool setting and did not extend to organized sports participation. For some families, increased ball-based activities in preschool could reduce the perceived need for leisure time sport in the short term. An analysis of the collaboration between sports clubs, preschools and municipalities showed that it was positively received by all involved but did not immediately result in new memberships in the short run. However, it potentially fostered lasting partnerships and inspired new joint initiatives, laying a solid foundation for sustainable, cross-sector cooperation in the future (31).

Despite the lack of short-timeeffects on formal sports participation, the intervention demonstrated positive effects on children's motor skills in a related study of the same population.^4^ Interviews with preschool teachers and observational visits indicated that the program and implementation support strategies fostered more frequent practice opportunities.^2^ However, variability across preschools in implementation fidelity and adoption likely diluted the overall impact.

While most similar studies focus on motor competences and/or mental and physical health status as outcomes, a few longitudinal studies provide valuable insights into how structured physical activity programs may influence children's engagement in organized sports. In Finland, Meklin et al. (21) followed 6-year-old children over 3 years and found that early participation in ball sports predicted higher perceived motor competence at age nine, particularly among girls. However, participation in multiple sports was correlated with lower perceived competence, indicating that overexposure or pressure may hinder long-term engagement. Parental support emerged as a key factor in maintaining sports club involvement.

As part of our collaboration program, eight site visits were conducted to introduce children to the offerings of local sports clubs. However, these efforts resulted in minimal formal enrollment, suggesting that increased awareness and experiential exposure alone may be insufficient to overcome logistical barriers or shift underlying dynamics of supply and demand. On the supply side, clubs’ outreach efforts varied in their ability to engage and accommodate newcomers in this age group. A notable success was a basketball club that, through the collaboration, established a new team for the age group they had not previously engaged in the club (31). On the demand side, factors such as children's existing leisure activity commitments, parental priorities, and the perceived relevance of the offerings played a significant role. In some cases, clubs may not have sufficiently adapted their activities to attract new participants, while many children were already involved in other sports. Furthermore, the local differences in participation (Table 2) highlight the importance of contextual factors, including socioeconomic conditions and the presence of well-established clubs with long-standing programs for young children.

Our cross-sectional analyses revealed both socioeconomic and cultural-based differences. Children from higher-income and sport-experienced households exhibited greater BPI scores and higher odds of sports participation. These findings echo wider international evidence of cultural skew in early-childhood activity offerings (26). Similarly, a national survey in Denmark showed relationship between 3 and 6 years children's sports participation and their parents' current sports participation and education length (24). In contrast to previous studies that focus on parents' current activity levels (24, 34, 35), this study employed a measure of overall sport experience to capture underlying cultural norms and values. This approach aligns with research on parental perceptions, suggesting that perceived benefits of sports participation may be shaped by parents' own sport backgrounds (19, 26). Interestingly, the proxy for family Danish nativity, language spoken at home, where associated with lower BPI and lower odds of ball-based sports participation, though not significant.

Being a girl was associated with lower BPI and reduced participation in ball-based sport, although participation in any organized sport did not differ significantly between girls and boys. Gender stereotyping in early years sport has been reported in the literature (20), and a previous study conducted in Denmark also identified cultural gender differences related to ball-based activities (18). It revealed a gendered dimension in soccer participation, shaped by parental expectations and societal norms, and emphasized the importance of inclusive messaging to ensure that girls feel equally welcome in sports clubs.

### Methodological strengths and limitations

Overall, the study employed a collaborative approach in developing the intervention, which included workshops with experts and key stakeholders. The intervention is among the first to actively build bridges between preschools and sports clubs, involving municipal administrators as collaborating actors. It was designed with a strong emphasis on sustainability, grounded in practical realities, and co-developed with practitioners to ensure that implementation could continue independently without relying on researchers as facilitators.

The study employed a non-randomized, wait-list control design across eight preschools, offering real-world insights, but findings are limited in generalizability. First of all, due to the recruitment procedure of the municipalities and preschools and secondly, due to the low response rate despite *in-situ* data collection support. Representativity of the participating parents can therefore be questioned. Furthermore, the wide confidence intervals observed in several analyses, particularly for the interaction term in the impact analysis, indicate substantial uncertainty in the estimates. The study was designed and powered to detect changes in motor competence rather than sports participation and was likely underpowered for these outcomes.

Furthermore, reliance on parent-reported outcomes introduces potential recall and social desirability biases, as well as proxy bias—where the parent's perceptions may not fully reflect the child's own experiences, preferences, or competences. Cultural and linguistic bias should also be mentioned due to almost a third of respondents speaking other languages at home with large differences between preschools. The questions used were tested in a native Danish test population but were not tested in English version or with participants having different cultural backgrounds.

### Implications for future studies and practice

The study provides a systematic foundation for identifying and refining key components of the intervention, its implementation processes, and the data collection strategy, supporting future adaptation, scalability, and transfer to other settings. While no immediate effects were observed on participation in organized sport, the results point to the potential value of strengthening foundational elements within preschool settings. As reflected in the intervention model, daily ball play is supported by a positive iterative process in which participation, experience, and competence reinforce each other. From this perspective, early ball play experiences may contribute to children's foundation for later engagement in physical activity and organized sport. Future interventions should therefore focus on sustaining these processes and strengthening connections between preschools, families, and community sports clubs. Four specific areas are highlighted for further development and qualification:

Differences in sports participation across preschools, along with variations in clubs' capacity, indicate that future initiatives should consider both the ability of sports clubs to welcome new children and the existing activity levels within the target group. While this study was anchored in the preschool setting, future efforts may benefit from adopting a stronger perspective of voluntary sports clubs as setting for capacity development.

Furthermore, although the intervention focused specifically on ball-based play and activities, a broader approach encompassing diverse forms of organized sport could mitigate capacity issues. Central to future development is a continued emphasis on equity, ensuring that all children—not only those from resource-rich backgrounds—are given early and meaningful opportunities to engage in sport. This includes targeted attention to girls, children with disabilities and children from less advantaged areas, where varied sport supply is limited.

The collaboration with sports clubs may support the implementation of ball-based pedagogical practices within preschools. Evidence from accompanying process evaluations suggests that the collaboration was positively received and contributed to increased focus on ball-based activities in everyday practice^2^ (31). However, its impact on children's leisure-time participation was limited. To effectively support engagement outside of institutional settings, greater parental involvement and high attention to structural, cultural, and economic barriers are essential.

Finally, methodological considerations should be addressed in future studies. The low response rate in this study highlights the need for robust mixed-methods evaluations that could combine quantitative enrollment data with qualitative interviews to uncover nuanced barriers and facilitators. Additionally, if parent-based questionnaires are used it should be tested for language-related differences by engaging parent from diverse backgrounds.

## Conclusion

The *Ball-based Play in Preschool* study highlights the multifaceted challenges of promoting organized sports participation in early childhood. While a separate study of the same intervention showed improvements in ball-related motor skill development, the current study found no short-time impact on formal sports enrollment at follow-up. Marked differences in sports participation and children's affinity for ball games across preschools raise important considerations for future intervention design. While nearly one-third of children in the intervention group engaged in daily ball-based play at home by program end—compared to 12% in the control group—it remains concerning that one third reported playing ball less than weekly. While many parents on behalf of their child reported high confidence and enjoyment in ball-based play, future interventions should pay particular attention to those who are less experienced or less confident.

Future initiatives may benefit from adopting the perspective of voluntary sports clubs as a starting point, broadening the scope beyond ball-based activities, and ensuring inclusive communication strategies.

## Data Availability

The raw data supporting the conclusions of this article will be made available by the authors, without undue reservation.

## Footnotes

^1^Grandt V, Pedersen MRL, Klein-Wengel TT, Meiner CB, Pedersen CP, Christiansen LB, et al. Motor skills in early childhood: exploring the roles of individual and family factor in Danish preschools. *Eur J Sport Soc*. [in review].

^2^Klein-Wengel TT, Nielsen JV, Pedersen MRL, Larsen LR, Larsen MN, Bertelsen K, et al. Getting the ball rolling – adoption and feasibility of ball-based play in preschools. *Glob Implement Res Appl*. [in review].

^3^Klein-Wengel TT, Nielsen JV, Skovgaard T, Pedersen MRL, Larsen MN, Kristensen MK, et al. Supporting implementation in preschool–based movement interventions: the role of research–based implementation support practitioners. *Health Promot Int*. [in preparation].

^4^Larsen MN, Grandt V, Pedersen MRL, Meiner CB, Klein-Wengel TT, Christiansen LB. Exploring effects on motor skills of the “Ball-based play in preschools” intervention: a stepped wedge case-controlled pilot study. Pilot and Feasibility Studies [in review].

## Supplementary material

Data Sheet1 Supplementary Figures S1-S3.

Data Sheet2 Activity card examples.
