# Supplementary figures and images for: Promoting ball-based play and sports participation for preschool children
Source: Front Sports Act Living. 2026 Jun 10;8:1815686. doi: 10.3389/fspor.2026.1815686 (PMC13291895; doi:10.3389/fspor.2026.1815686)

# Supplementary Figure 1-3

Response distributions of three questions in the Ball Play Index

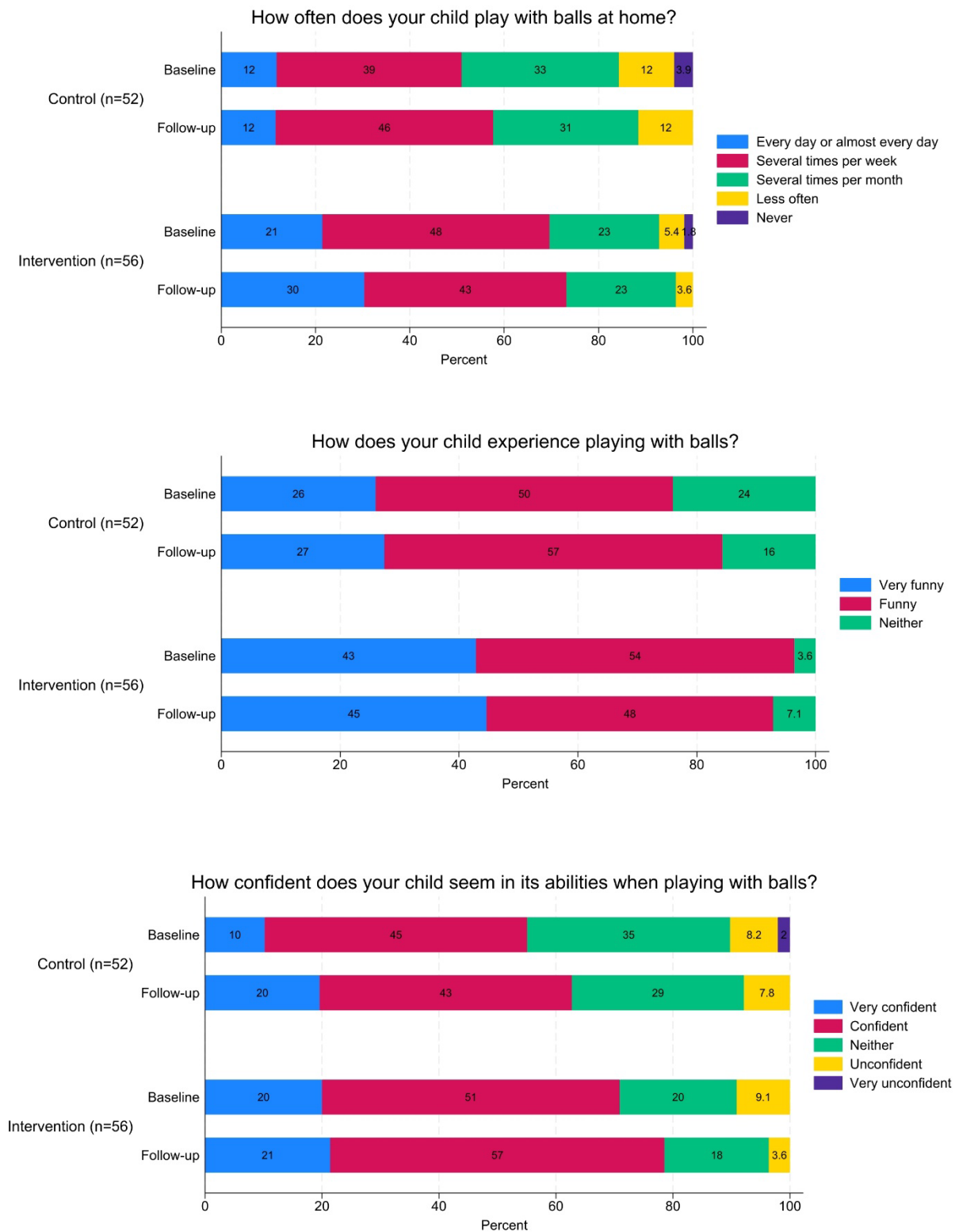

Supplement: Data Sheet1 — Supplementary Figures S1-S3. [file Datasheet1.pdf]
