# Supplementary material for: Promoting ball-based play and sports participation for preschool children
Source: Front Sports Act Living. 2026 Jun 10;8:1815686. doi: 10.3389/fspor.2026.1815686 (PMC13291895; doi:10.3389/fspor.2026.1815686)

AGILITY COURSE WITH BALLS

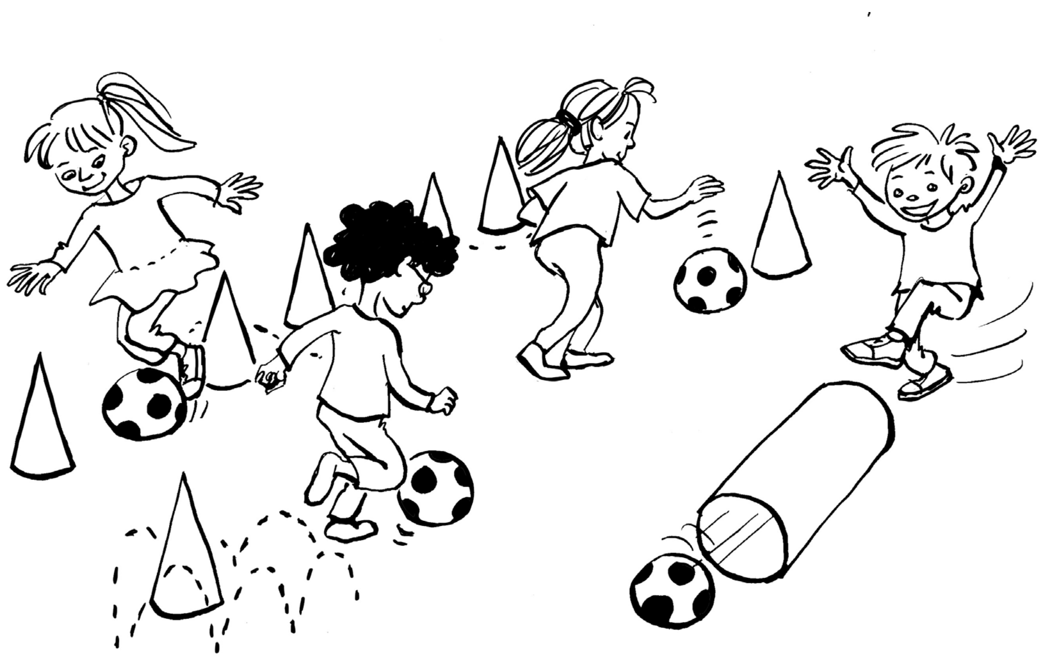

Fundamental motor skills

AGILITY COURSE WITH BALLS

BALL-BASED PLAY IN PREESCHOOLS

PRACTICAL INFORMATION/EQUIPMENT	VARIATIONS
Balls and/or beanbags.<br><br>Equipment for setting up an agility course where the ball can be incorporated.	The children can help decide how to move, how to use the balls, or how the course can be set up.<br><br>The children can work in pairs and help each other through the course.
**ACTIVITY DESCRIPTION**	**POINTS OF ATTENTION**
The children move around the course, either carrying a ball throughout the entire course or completing ball-related tasks at specific points along the way.<br><br>Elements that can be included are, e.g., slalom running, jumping, hopping, going over, under or through obstacles, somersaults, hitting a target ...	Make sure all children are moving.<br><br>Allow room for creativity and initiative.<br><br>Work with a range of fundamental motor skills.

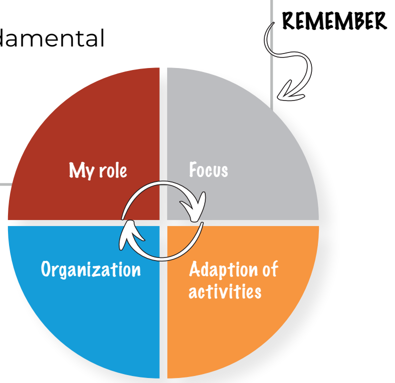

HIT THE BALLOON

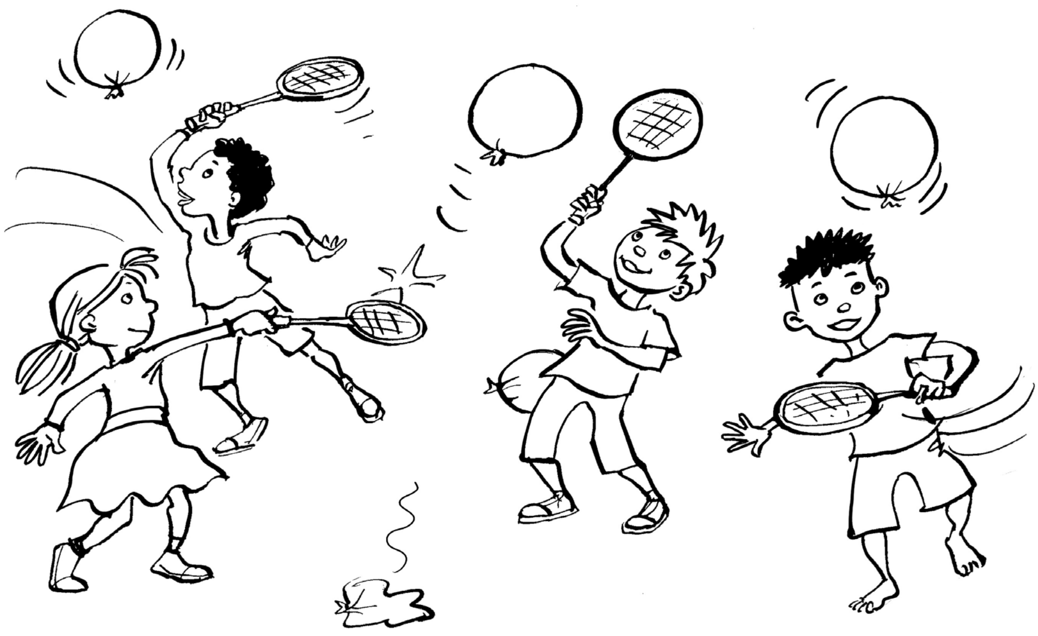

Ball exploration

HIT THE BALLOON

BALL-BASED PLAY IN PREESCHOOLS

PRACTICAL INFORMATION/EQUIPMENT	VARIATIONS
Balloons and fly swatters.	The children can transport the balloons from A to B by hitting them with the fly swatter.<br><br>The balloons can be hung from the ceiling with a string, and the children must hit them while they are hanging there.<br><br>The children can work in pairs and pass the balloon to each other.<br><br>The children can use their hand instead of the fly swatter.<br><br>Use soap bubbles.
**ACTIVITY DESCRIPTION**	**POINTS OF ATTENTION**
Each child is given a balloon and a fly swatter, and they must keep the balloon in the air by hitting it with the fly swatter.	Allow room for creativity and initiative.<br><br>Be aware of flying balls.<br><br>Join in the activity yourself.

REMEMBER

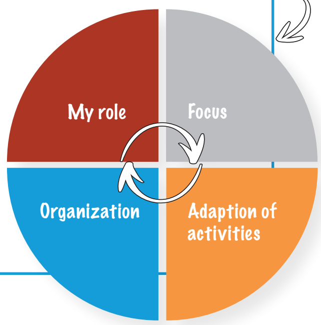

THE BOMB

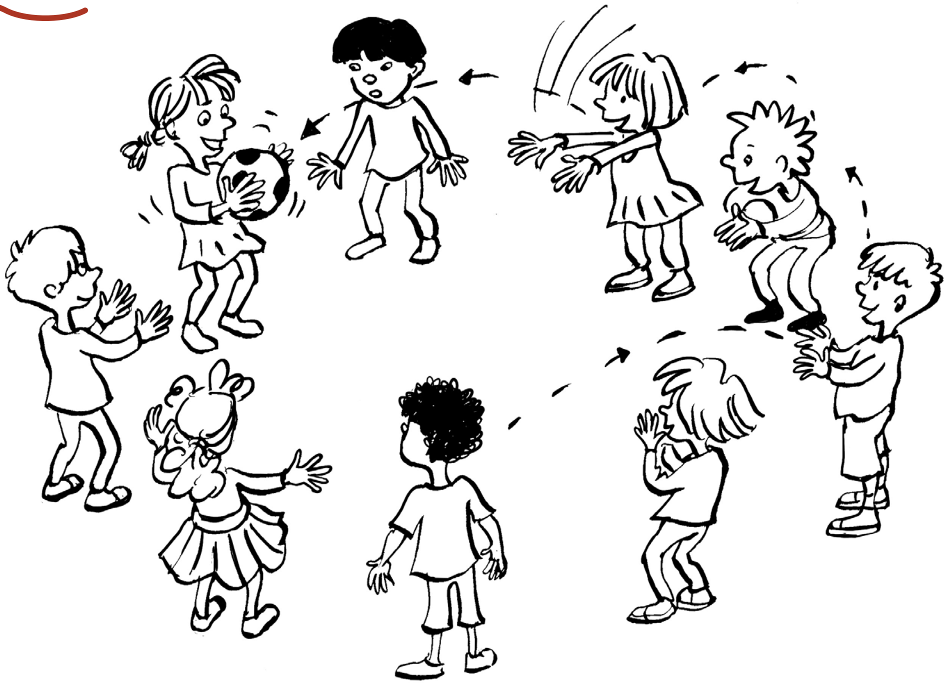

Playing together

THE BOMB

BALL-BASED PLAY IN PREESCHOOLS

PRACTICAL INFORMATION/EQUIPMENT

Ball(s).

VARIATIONS

You can add more balls and possibly different tasks. E.g., the yellow ball must be passed to the person next to you, while the red ball must be passed to someone else.

ACTIVITY DESCRIPTION

Everyone stands in a circle and passes the bomb (the ball) around without dropping it – if it is dropped, it explodes.

The ball can be passed in different ways, e.g., by handing it to the person next to you, throwing it to the person next to you, or throwing it to someone other than your neighbour.

If the bomb explodes (the ball is dropped), the adult can give everyone a task, e.g., jumping 10 times or rolling, after which the bomb is ready again.

POINTS OF ATTENTION

Work with a range of fundamental motor skills.

Support the children in their cooperation.

Join in the activity yourself.

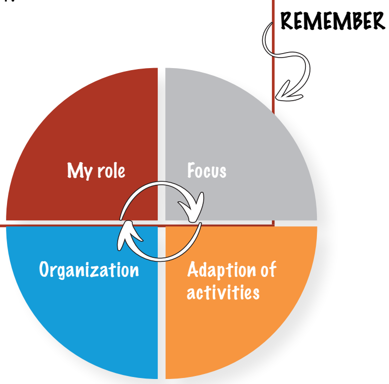

THE GOLD HUNT

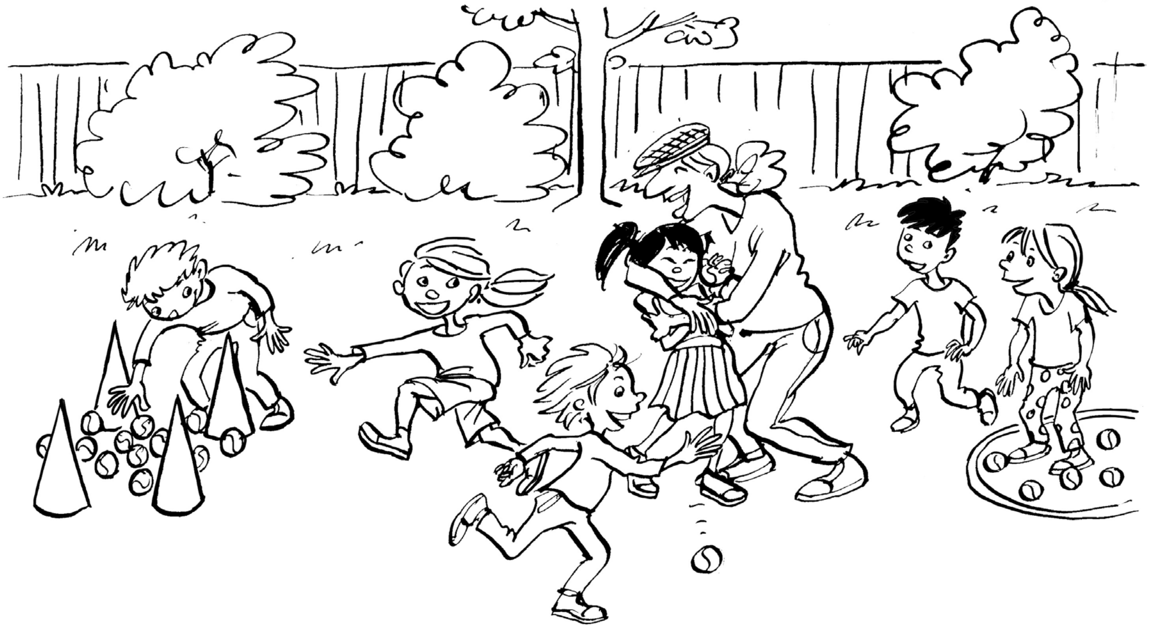

Playing against someone

THE GOLD HUNT

BALL-BASED PLAY IN PREESCHOOLS

PRACTICAL INFORMATION/EQUIPMENT

Balls.

Optional equipment: cones, hula hoops, chalk.

Make use of the surroundings (indoors and outdoors).

VARIATIONS

If there are multiple teams and starting points, the children can steal gold from each other once the mine has been emptied.

It can also be played as two teams against each other, e.g., three against three, where the two teams try to steal gold from the other team's starting point. Each team could then have one catcher and two runners.

The robber can have a soft foam ball that can be thrown at the gold diggers.

ACTIVITY DESCRIPTION

The children must collect balls from the gold mine and bring them back to their starting point without being caught by a robber (an adult). If a child is caught, they may have to drop the ball and return to the starting point.

The transport can be done in different ways, e.g., running while holding the ball, dribbling with the feet, or dribbling with the hands. There can be one team – or several teams with multiple starting points.

POINTS OF ATTENTION

Adapt the challenges to the children..

Join in the activity yourself.

Ensure that the children experience an appropriate level of challenge.

REMEMBER

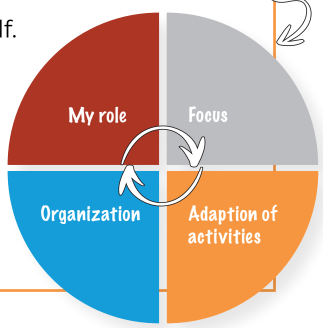

Supplement: Data Sheet2 — Activity card examples. [file Datasheet2.pdf]
